# Insulin-Like Growth Factor-1 as a Possible Alternative to Bone Morphogenetic Protein-7 to Induce Osteogenic Differentiation of Human Mesenchymal Stem Cells in Vitro

**DOI:** 10.3390/ijms19061674

**Published:** 2018-06-05

**Authors:** Bruno Reible, Gerhard Schmidmaier, Arash Moghaddam, Fabian Westhauser

**Affiliations:** 1 HTRG—Heidelberg Trauma Research Group, Center of Orthopedics, Traumatology, and Spinal Cord Injury, Heidelberg University Hospital, Schlierbacher Landstraße 200a, 69118 Heidelberg, Germany; bruno.reible@med.uni-heidelberg.de (B.R.); gerhard.schmidmaier@med.uni-heidelberg.de (G.S.); arash.moghaddam@klinikum-ab-alz.de (A.M.); 2 ATORG—Aschaffenburg Trauma and Orthopedic Research Group, Center for Trauma Surgery, Orthopedics, and Sports Medicine, Klinikum Aschaffenburg-Alzenau, Am Hasenkopf 1, 63739 Aschaffenburg, Germany

**Keywords:** human mesenchymal stem cells (MSC), Insulin-like Growth Factor-1 (IGF-1), Bone Morphogenetic Protein-7 (BMP-7), osteogenic differentiation, in vitro

## Abstract

Growth factors and mesenchymal stem cells (MSC) support consolidation of bone defects. Bone Morphogenetic Protein-7 (BMP-7) has been used clinically and experimentally, but the outcomes remain controversial. Increased systemic expression of Insulin-like Growth Factor-1 (IGF-1) significantly correlates with successful regeneration of bone healing disorders, making IGF-1 a promising alternative to BMP-7. There is no experimental data comparing the osteoinductive potential of IGF-1 and BMP-7. Therefore, in this study, the influence of IGF-1 and BMP-7 in different concentrations on the osteogenic differentiation of two human MSC-subtypes, isolated from reaming debris (RMSC) and iliac crest bone marrow (BMSC) has been assessed. A more sensitive reaction of BMSC towards stimulation with IGF-1 in concentrations of 400–800 ng/mL was found, leading to a significantly higher degree of osteogenic differentiation compared to stimulation with BMP-7. RMSC react more sensitively to stimulation with BMP-7 compared to BMSC. Lower concentrations of IGF-1 were necessary to significantly increase osteogenic differentiation of RMSC and BMSC compared to BMP-7. Therefore, IGF-1 should be considered as a valuable option to improve osteogenic differentiation of MSC and merits further experimental consideration. The MSC subtype and method of differentiation factor application also have to be considered, as they affect the outcome of osteogenic differentiation.

## 1. Introduction

Modern orthopedic surgery is still facing consolidation of critical-sized bone defects as one of the major clinical problems [1]. Amongst others, the well-orchestrated use of growth and differentiation factors in combination with autologous cells is amongst the key factors to achieve successful consolidation [2,3].

Most experimental approaches to optimize the interaction of cells and differentiation factors to facilitate bone healing are based on the use of human mesenchymal stem cells (MSC) [4,5]. Bone regeneration originates from MSC, which are able to differentiate into osteoblasts and are in charge of building new bone substance [6]. MSC can be isolated from various tissues: The most common sources are bone marrow, cartilage, and fat tissue [7]. Previous studies showed that there are differences in the osteogenic potential and in their reactivity to differentiation stimuli, depending on the origin of the isolated MSC [1,7,8]. MSC isolated from iliac crest bone marrow (BMSC) and from reaming debris (RMSC) show superior osteogenic potential compared to MSC harvested from other sources [7].

In clinical application, once mechanical stability is provided, bone defects are usually filled with bone harvested from the iliac crest—referred to as being the most popular and most widely used autologous tissue source [7,9]. However, RMSC have not only demonstrated a better osteogenic potency compared to BMSC in experimental settings, there is also more graft material to be harvested and complications are lower overall compared to iliac crest bone grafting [1,10,11]. Furthermore, the interaction of RMSC with growth and differentiation factors, such as Bone Morphogenetic Protein-7 (BMP-7), seems to work better in terms of stimulation of osteogenic differentiation compared to BMSC in experimental settings [1,8].

BMP-7 has been proven to stimulate bone formation in vitro and in vivo [8,12,13]. There is evidence that the application in a clinical setting leads to consolidation of bone defects, even in cases of delayed healing or non-union [14,15,16]. However, the clinical results are controversial: While some authors report positive effects of BMP-7 application on bone healing, other studies did not find a positive influence on bone defect consolidation when BMP-7 was used [14,17,18,19,20]. Using the currently available application form of collagen-incorporated BMP-7 applied to the defect site does not guarantee a continuous stimulation of the local cells by long-lasting BMP-7 release [21,22].

A previous study of our group using both BMP-7 and Insulin-like Growth Factor-1 (IGF-1) demonstrated that osteogenic differentiation of MSC depends on the method of stimulation: Continuous stimulation in vitro is necessary to sustainably enhance osteogenic differentiation of both RMSC and BMSC [23]. This might be transferable to the clinical situation and could therefore be a possible explanation for the controversial clinical findings. This previous study served as a pilot research for our current study, which uses similar methods to further investigate the dose-response relationship of stimulating RMSC and BMSC with IGF-1 and BMP-7.

Insulin-like Growth Factor-1 (IGF-1) is one of the key players not only in skeletal development and maintenance of the skeletal structures during life, but is also produced locally during callus formation and defect repair [24,25].

IGF-1 plays an important role during fetal organogenesis and was shown to facilitate regeneration of various tissues such as bone, muscle, or nerves [26,27,28,29,30,31]. The ability of IGF-1 to promote this regeneration of tissue is associated with a reduction in pro-inflammatory cytokines and a corresponding stimulation of anti-inflammatory cytokines [31]. Additionally, ghrelin, a growth hormone secretagogue produced mainly in the stomach, was shown to exhibit protective and regenerative effects in various tissues via an increase in the release of endogenous IGF-1 and growth hormone [32,33,34,35,36,37,38,39,40,41]. A recent study also found ghrelin promoted an increased expression of homeobox protein B4 in rat bone marrow stromal cells, which is a transcription factor involved in stem cells’ survival and regeneration [42].

The positive influence of IGF-1 on fracture healing has been demonstrated in several studies: Local administration of IGF-1 in a rodent model led to formation of mechanically stronger callus and was able to accelerate fracture healing [43,44]. In a sheep model, best consolidation rates in distraction osteogenesis were achieved by a combination of local autograft with local IGF-1-application [45]. Further in vivo studies showed that IGF-1 has a comparable effect to stimulation of callus formation compared to Bone Morphogenetic Protein-2 (BMP-2) [46,47]. Furthermore, IGF-1 is able to enhance osteogenic differentiation of MSC towards osteogenic precursor cells, such as osteoblasts [48,49].

Recent clinical trials demonstrated that IGF-1 plays an outstanding role in consolidation of delayed union: High serum levels of IGF-1 correlate with successful treatment outcomes [24,50,51]. These cases require biological stimulation much more than usual fractures. Therefore, the local use of IGF-1 at the defect site in these cases might be a promising alternative to the clinically established differentiation factors, such as BMP-7.

Whilst there is evidence about the use of BMP-7 in both clinical and experimental settings, there is no evidence concerning the effect of IGF-1 on osteogenic differentiation of RMSC and BMSC in vitro. Furthermore, IGF-1 and BMP-7 have never been compared in one and the same osteogenic differentiation protocol before. It is also unknown whether there is an “ideal” concentration of both IGF-1 and BMP-7 to provide optimal osteogenic stimulation of BMSC and RMSC in vitro. The use of differentiation factors requires the application of an optimal concentration which is not yet defined: Using too little stimulation is probably not sufficient to induce osteogenic differentiation, whilst the application of too high concentrations is uneconomic and possibly harmful, because of the increased risk of systemic and local side effects if applied in vivo or clinically [52,53]. Therefore, a general overview of the correlation of dose and effect should be assessed in vitro before possible transfer into other settings.

So far, basic experimental data is missing to analyze and confirm the osteoinductive potential of IGF-1 in comparison to BMP-7 on RMSC and BMSC depending on the applied concentration. Therefore, in this study, we compare the influence of continuous stimulation by IGF-1 and BMP-7 in different concentrations on the osteogenic differentiation of human BMSC and RMSC in vitro.

## 2. Results

### 2.1. Cellular Characteristics and Morphology

**Figure 1:**
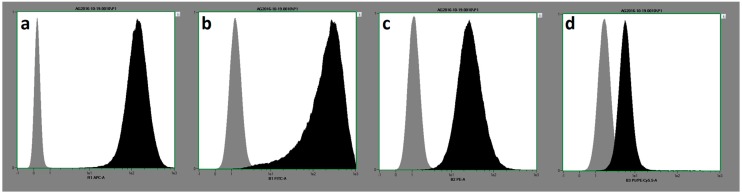
(**a**–**d**) show histograms from the flow cytometry, labelled with the surface proteins that were analyzed ((**a**) corresponds to CD73; (**b**) to CD90; (**c**) to CD 105; and (**d**) to the negative control composed of CD14, CD20, CD34, and CD45). The grey bar on the left of each graph shows the results of marking the MSC with an isotype control of murine antibodies against the respective surface proteins, the black bar on the right shows the results of the marking with human antibodies. The *x*-axis is a logarithmic scale of fluorescence intensity, the *y*-axis a height-normalized linear scale of cell number. The results are taken representatively from one donor in the collective.

**Figure 2:**
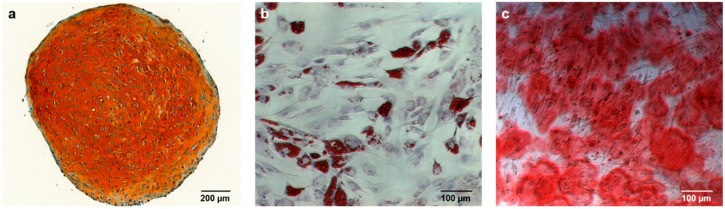
Representative analysis of trilineage differentiation in cells of one donor. Chondrogenic differentiation (**a**) is indicated by the orange extracellular matrix in Safranin O/Fast Green staining. The red-colored fatty vacuoles in Oilred-O staining (**b**) are correlates of adipogenic differentiation. Alizarin red stains extracellular calcium deposited by osteogenically differentiated mesenchymal stem cells (MSC) red (**c**).

Both subtypes of MSC, RMSC and BMSC, showed similar expression patterns of the relevant surface markers (Table 1, Figure 1), as well as the ability for trilineage differentiation. Representative qualitative results from osteogenic, chondrogenic, and adipogenic differentiation are shown in Figure 2. Quantitative results concerning osteogenic differentiation as the main part of this study are described within the following paragraphs.

### 2.2. Alizarin Red Staining

#### 2.2.1. Comparison to Control Group

**Figure 3:**
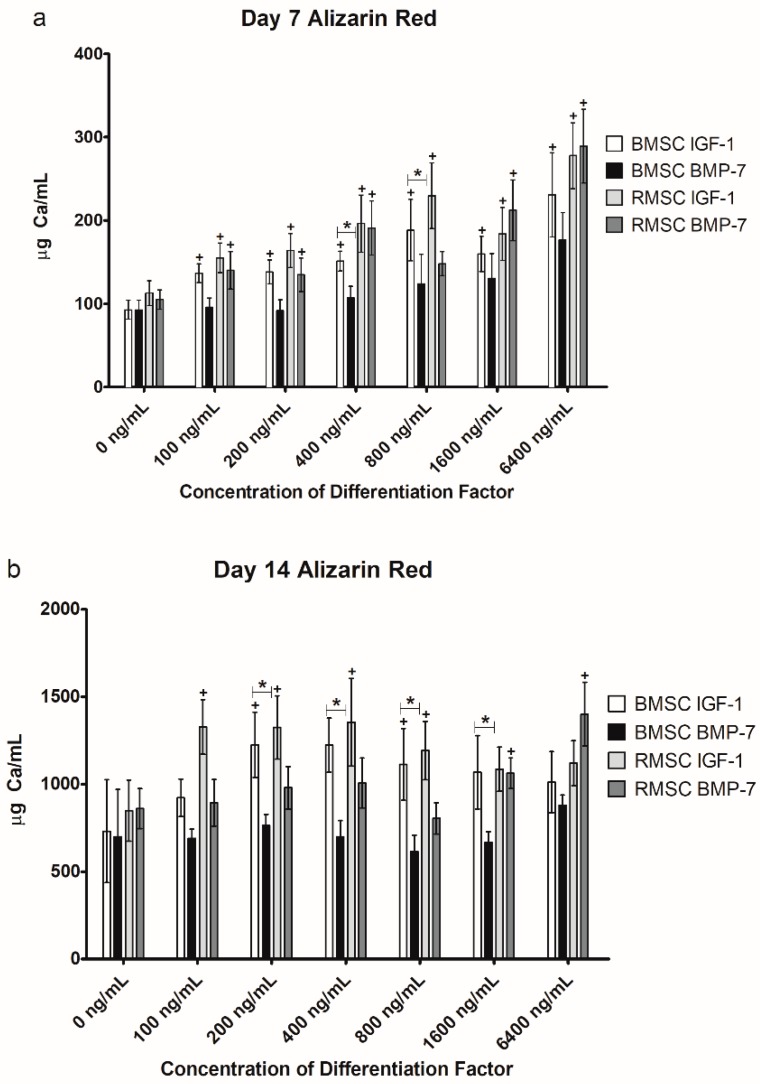
Results of the Alizarin Red staining, showing day 7 (**a**) and 14 (**b**) separately for increased clarity. Results are displayed as bar graphs with the standard error of the mean shown as whiskers above and below each bar. Results for iliac crest bone marrow (BMSC) stimulated with Insulin-like Growth Factor-1 (IGF-1) are always shown in the first bar, followed by BMSC with Bone Morphogenetic Protein-7 (BMP-7), reaming debris (RMSC) with IGF-1, and finally RMSC with BMP-7. * marks significant differences in the two adjacent bars underneath it, + marks significant differences between the bar underneath it and the corresponding unstimulated control group. All measurements were carried out in both biological and technical duplicates.

In order to determine whether the used differentiation factors had any effect on the cells in culture, cells differentiated under the influence of differentiation factors were first compared to cells that were grown without any additional supplements. The resulting outcomes of the Alizarin Red staining (determining the extracellular calcium deposit as a correlate of osteogenic differentiation) are outlined below (Figure 3).

BMSC showed significantly higher extracellular calcium deposition after seven days of stimulation (D7) with IGF-1 at all doses tested, in comparison to the unstimulated control group (Figure 3a). Compared to the control group, IGF-1 given for seven days at a concentration of 100, 200, 400, 800, 1600, and 6400 ng/mL increased calcium deposition in BMSC by 47.31% (*p* = 0.028), 49.09% (*p* = 0.028), 63.01% (*p* = 0.028), 103.09% (*p* = 0.046), 72.38% (*p* = 0.028), and 148.83% (*p* = 0.028), respectively. After 14 days in culture (D14), calcium deposition of BMSC was significantly higher compared to the control group (which was also differentiated for 14 days) for 200 ng/mL (67.69%, *p* = 0.047) and 800 ng/mL (52.40%, *p* = 0.046) of IGF-1. RMSC reacted similarly to BMSC to stimulation with IGF-1, with significant increases of calcium deposition being detectable after seven days as well, again compared to an unstimulated control group (an increase of 37.49% (*p* = 0.028), 45.43% (*p* = 0.028), 74.07% (*p* = 0.046), 103.67% (*p* = 0.028), 63.12% (*p* = 0.028), and 146.41% (*p* = 0.028) for 100, 200, 400, 800, 1600, and 6400 ng/mL IGF-1). Compared to BMSC, after 14 days, significant increases in calcium deposition happened earlier with RMSC, being observable for all tested concentrations of IGF-1, except for 1600 and 6400 ng/mL (an increase of 56.37% (*p* = 0.028), 56.08% (*p* = 0.028), 59.54% (*p* = 0.028), and 40.51% (*p* = 0.028) for 100, 200, 400, and 800 ng/mL IGF-1, respectively).

For BMP-7, all measured values were again compared to a control group of cells not under the influence of any additional stimulation factors but also differentiated for 7 and 14 days, respectively. In the BMSC group, no concentration of BMP-7 yielded significant increases in calcium deposit, either on D7 or D14. On D7, RMSC showed a significant increase of calcium deposition for all concentrations except 800 ng/mL (an increase of 33.45% (*p* = 0.046), 28.35% (*p* = 0.028), 81.88% (*p* = 0.028), 102.28% (*p* = 0.028), and 175.48% (*p* = 0.046) for 100, 200, 400, 1600, and 6400 ng/mL BMP-7). RMSC seem to react more sensitively to stimulation with BMP-7 compared to BMSC.

After 14 days in differentiation, significant increases in calcium deposition could be detected for RMSC stimulated with 1600 (23.40%, *p* = 0.028) and 6400 ng/mL BMP-7 (62.72%, *p* = 0.028).

#### 2.2.2. Comparison of IGF-1 to BMP-7

As soon as biological activity of the differentiation factors was affirmed, the results of the IGF-1 group were compared to those of the BMP-7 group. These results are outlined in Figure 3 as well.

A significantly higher grade of differentiation was found for IGF-1 compared to BMP-7 for BMSC on day 7 at 400 and 800 ng/mL (*p* = 0.041 in both cases; Figure 3a), as well as on day 14 at 200 (*p* = 0.015), 400 (*p* = 0.026), 800 (*p* = 0.026), and 1600 (*p* = 0.041) ng/mL (Figure 3b). Stimulation with IGF-1 resulted in non-significantly higher differentiation for BMSC at all other concentrations as well. No significant differences were found for RMSC.

Although not a main part of this study, when comparing the results of stimulating RMSC with BMP-7 to BMSC stimulated with BMP-7, a significantly higher calcium deposit was found for RMSC stimulated with concentrations of 400 and 1600 ng/mL of BMP-7 (77.98% (*p* = 0.026) and 62,71% (*p* = 0.041) higher, respectively) on day 7, as well as for 1600 and 6400 ng/mL of BMP-7 (58.94% (*p* = 0.015) and 59.10% (*p* = 0.026) higher) on day 14. Comparing the results of stimulation with IGF-1 in a similar way yielded no significant differences, although the calcium deposit was uniformly higher for RMSC again. This again seems to underline the more sensitive reaction of RMSC to BMP-7 compared to BMSC.

### 2.3. Alkaline Phosphatase (ALP) Activity

Along with extracellular calcium deposition, measured by Alizarin Red staining, the activity of alkaline phosphatase (ALP) correlates with osteogenic differentiation of MSC. ALP is produced and partially released by osteoblasts and osteoblast precursors [23].

#### 2.3.1. Comparison to Control Group

**Figure 4:**
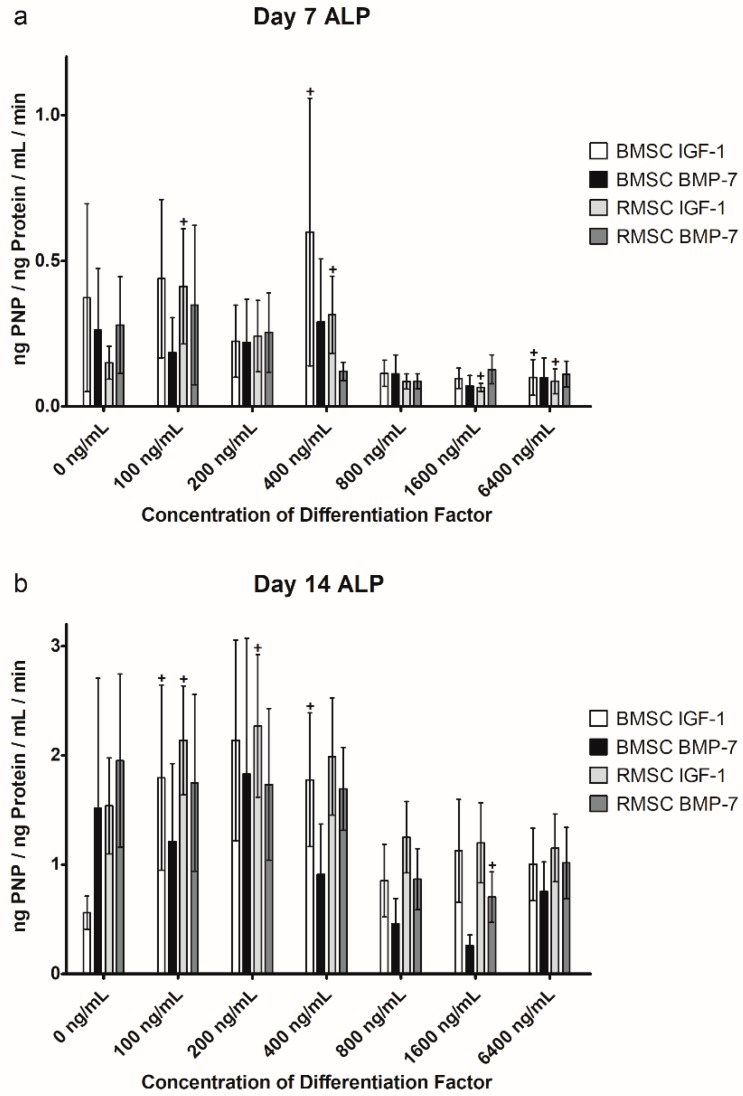
Comparison of the alkaline phosphatase (ALP) activity between MSC stimulated with IGF-1 or BMP-7, again showing day 7 (**a**) and 14 (**b**) separately for increased clarity. Results are displayed as bar graphs with the standard error of the mean shown as whiskers above and below each bar. Results for BMSC stimulated with IGF-1 are always shown in the first bar, followed by BMSC with BMP-7, RMSC with IGF-1, and finally BMSC with BMP-7. (+) marks significant differences between the bar underneath it and the corresponding unstimulated control group. All measurements were carried out in both biological and technical duplicates. The unit of ALP activity is ng para-Nitrophenol (PNP)/ng Protein/mL/min.

Again, a comparison to the control group was performed to verify biological activity, as well as to find at which concentration the differentiation factors started to have an effect on ALP activity in the cells. The results are shown in Figure 4.

In the BMSC group stimulated with IGF-1, ALP activity increased significantly on D7 at a concentration of 400 ng/mL (60.47%, *p* = 0.028), but showed a significant decrease at 6400 ng/mL (−73.21%, *p* = 0.046) as well, compared to an unstimulated control group. After 14 days of differentiation with IGF-1, ALP showed increased activity compared to the control group for all tested concentrations, with significant differences for 100 ng/mL (220.75%, *p* = 0.046) and 400 ng/mL (217.26%, *p* = 0.046). However, when BMSC were stimulated with BMP-7, mostly a decrease of ALP activity was detectable after both 7 and 14 days of differentiation, with a significant difference on D14 at 1600 ng/mL (−83.07%, *p* = 0.028).

When stimulated with lower concentrations of IGF-1, RMSC showed increased ALP activity after 7 and 14 days, with significant increases on D7 at 100 (174.08%, *p* = 0.028) and 400 ng/mL (109.43%, *p* = 0.028), as well as 100 (39.05%, *p* = 0.028) and 200 ng/mL (47.66%, *p* = 0.046) on D14. When higher concentrations were applied (starting from 800 ng/mL), ALP activity decreased compared to the unstimulated cells, with significant decreases at 1600 (−56.36%, *p* = 0.046) and 6400 ng/mL (−42.32%, *p* = 0.046) on D7. For BMP-7, with the exception of 100 ng/mL on D7, ALP activity in RMSC decreased uniformly across all observed concentrations, with a significant decrease on D14 at 1600 ng/mL (−64.05%, *p* = 0.028).

#### 2.3.2. Comparison of IGF-1 to BMP-7

The same calculations performed on the results of the Alizarin Red staining were also performed on the results of the ALP activity measurement to find any differences in differentiation between the two differentiation factors used. The outcomes are shown above in Figure 4.

In the ALP measurement, no significant differences between stimulation with IGF-1 or BMP-7, or between RMSC and BMSC could be found.

## 3. Discussion

Bone defect treatment remains one of the major challenges in orthopedic surgery. However, modern treatment options have been developed combining the use of cells, growth factors, and bone substitutes [2,8]. In patients showing successful consolidation of bone defects, a high expression of IGF-1 in peripheral serum was observed and BMP-7 underwent extensive use in clinical routine and experimental approaches, making these factors attractive for further experimental consideration [20,24,50]. The aim of this study was to compare the influence of IGF-1 and BMP-7 in multiple concentrations on the osteogenic differentiation of human BMSC and RMSC in vitro.

It has been shown that MSC stimulated by either IGF-1 or BMP-7 exhibited significantly better osteogenic differentiation in vitro and in vivo, compared to unstimulated control groups [1,8,23,48,49,54,55]. Our group demonstrated in a pilot study that continuous stimulation of MSC with IGF-1 and BMP-7 is necessary to significantly influence osteogenic differentiation [23]. The pilot study focused on the importance of stimulation length, but also showed interesting results regarding the possible use of IGF-1 as a potent stimulation factor for osteogenic differentiation. However, it did not include a sufficient range of different concentrations of stimulation factor to facilitate a proper dose-response analysis, and apart from this pilot study, these factors have never been compared directly to each other in different concentrations, and the effects of the applied concentrations of growth factors on osteogenic differentiation still remain unclear. Positive aspects of the study are the use of MSC of human origin as well as the structured comparison of parallel concentrations of IGF-1 and BMP-7, providing directly comparable data, and the use of well-established methods: Correlates of osteogenic differentiation of RMSC and BMSC were analyzed by measurement of ALP activity and by determination of extracellular calcium deposition after 7 and 14 days in osteogenic differentiation [7]. A limitation of this study is the low number of donors, which could cause a comparably lower number of statistically significant differences between the different groups. Also, the use of additional methods, such as measurement of specific bone markers, for example, osteocalcin or Runt-related transcription factor 2 (RunX2) or quantitative real-time PCR could strengthen the findings of this study and should be considered for further studies [56].

Our results reveal that superior osteogenic differentiation of BMSC can be achieved by stimulation with average concentrations of IGF-1 of about 400 ng/mL, verified by significant increases in extracellular calcium deposition after 7 days, as well as a significant increase in ALP activity after both 7 and 14 days. BMSC react more sensitively to stimulation with IGF-1, compared to stimulation with BMP-7, which, in our experiment, did not lead to significant increases in differentiation at any tested concentration. Low and medium concentrations of IGF-1 led to significantly increased ALP activity in both BMSC and RMSC on D7, as well as D14, but higher concentrations caused a decrease in ALP activity compared to the control group (except for BMSC stimulated with IGF-1 on D14). BMP-7, on the other hand, caused a decrease in ALP activity compared to the control group across almost all tested concentrations, cell types, and time points. However, except for 1600 ng/mL on D14 for both BMSC and RMSC, those decreases were not significant.

RMSC show a similar reaction to stimulation with IGF-1, but react more sensitively to stimulation with BMP-7 compared to BMSC. Parallel findings were obtained in recent studies of our group, showing that RMSC react more sensitively to stimulation with BMP-7 compared to BMSC in vivo [1,8]. So far, the reasons for the differences in cellular characteristics and the response to differentiation stimuli induced by growth factors remain unclear. The phenotypes of both MSC-subtypes used in this study are identical: The cells show the same expression of the MSC-defining surface characteristics (Table 1, Figure 1) [57]. RMSC and BMSC are derived from the same tissue (bone marrow), but from different parts of the skeleton (diaphysis of the femur vs. iliac crest) [7]. Studies using adipose-tissue derived MSC (ATSC) showed that regardless of phenotype, the osteogenic differentiation properties are lower compared to BMSC and RMSC [7]. Therefore, even when cells show the typical morphological characteristics of MSC there still must be some cellular differences remaining: One possible explanation amongst several others could be found in different cellular surface receptor characteristics and/or density. Higher expression of receptors providing interaction with BMP-7 and IGF-1 on the MSC surface may lead to a more efficient transduction of differentiation stimuli into the cell [5,23]. Further studies should characterize the different MSC subtypes more precisely to provide a better understanding of the cellular differences.

The performance of the used differentiation factors in direct comparison revealed that IGF-1 induces strong osteogenic differentiation in BMSC, even in low concentrations and in the small collective used for this study. The same concentrations resulted in superior differentiation in RMSC as well. An optimal range of differentiation factor concentration in the presented experimental setting may be 400–800 ng/mL. Within this range, compared to the control group, a significant improvement of extracellular calcium deposit, as well as a significant increase in ALP activity, is achieved. Furthermore, IGF-1 caused significantly higher calcium deposition, as well as non-significantly higher levels of ALP activity in this range, compared to BMP-7. When using higher concentrations, negative effects are observed regarding decreasing ALP activity, as discussed above.

Whilst RMSC react more sensitively than BMSC to stimulation with BMP-7, slightly higher absolute concentrations of BMP-7 are necessary to induce a significant increase of osteogenic differentiation compared to IGF-1. BMSC did not react with a significant increase of osteogenic differentiation to stimulation with BMP-7 at all in our experiments. Whilst the use of high doses of BMP-7 is reported to be safe in experimental and clinical approaches, the need for higher local concentration leads not only to increased costs, but also to an increased theoretical risk for dose-associated mid- and long-term side effects [19,58,59]. Our results are supported by findings of previous studies, revealing that very high concentrations of BMPs tend to have a negative effect on osteogenic differentiation caused by a BMP-induced negative-feedback loop [21].

Therefore, IGF-1, being more effective in lower doses, should be considered as a potential option to enhance bone formation in further experimental approaches. Studies providing additional in vitro and in vivo data about the efficiency and safety of local IGF-1 application should be conducted.

In a recent pilot study, our group showed that osteogenic differentiation differs significantly depending not only on the concentration, but also on the mode of growth factor application. Short-term stimulation by IGF-1 and BMP-7 at the very beginning of the differentiation period for only 24 h is not sufficient to induce superior osteogenic differentiation compared to a group of MSC that have been continuously stimulated by these differentiation factors [23]. These findings are of significant relevance for the use of differentiation factors, also in context with the findings from this study: A delivery method has to be found providing continuous release of differentiation factors in an optimal dose. Based on the findings, IGF-1 should be used in upcoming studies, for example as part of bone substitutes or incorporated into appropriate carriers providing a stable release of IGF-1 in an adequate concentration to the surrounding cells to properly induce osteogenic differentiation and subsequently bone formation.

## 4. Materials and Methods

### 4.1. Donor Patients

RMSC and BMSC of six donors (1 female, 5 male) with an average age of 60.8 years (median 58.5, range 46 to 76) were harvested as described below. To obtain a realistic reflection of clinical routine, the donors included in this study were selected randomly without matching for clinical risk factors, weight, sex, and/or age.

The donors were treated for long bone non-unions at Heidelberg University Hospital and gave informed consent to participate in this study. The study was approved by the ethics committee of the University of Heidelberg (S-443/2015) according to the declaration of Helsinki in its present form. The study was conducted in accordance with the European and national regulations concerning the use of human donor material in experimental studies.

### 4.2. Primary Cell Culture and Isolation of Mesenchymal Stem Cells

Harvesting of primary material, primary culture, and isolation of RMSC and BMSC was conducted according to protocols published previously [1,7,23].

The isolated cells were defined as mesenchymal stem cells according to the criteria defined by Dominici et al., meaning plastic adherence, trilineage differentiation, and surface characterization as described previously [23,57]. In short, surface characteristics were defined by flow cytometry (FACS) analysis, demanding that >95% of the isolated cells detected positive for CD90, CD73, and CD105 and a maximum of 2% of the cells may be positive for CD14, CD20, CD 34, and CD45. Analysis was performed with a MACSQuant^®^ Analyzer 10 (Miltenyi Biotec, Bergisch Gladbach, Germany) using the MSC Phenotyping Kit (Miltenyi Biotec) according to the manufacturer’s instructions (Figure 1).

For evaluation of trilineage differentiation, cells were cultivated in respective differentiation media as follows: Cells were cultivated in chondrogenic differentiation medium (Dulbecco’s modified eagle medium (DMEM) high glucose (Thermo Fisher Scientific, Dreieich, Germany), 0.1 μM dexamethasone, 0.17 mM ascorbic acid 2-phosphate, 5 μg/mL transferrin, 5 ng/mL selenin, 1 mM sodium pyruvate, 0.35 mM proline, 1.25 mg/mL bovine serum albumin (BSA) (all Sigma-Aldrich, Steinheim, Germany), penicillin/streptomycin 100 mg/L (Merck, Darmstadt, Germany), amphotericin B 2.5 mg/L (Merck), 0.1375 IE/mL insulin glargin (Sanofi-Aventis, Frankfurt am Main, Germany), and 10 ng/mL Transforming growth factor β1 (TGF-β1) (Miltenyi Biotec) for 42 days to induce the formation of chondrogenic spheroids. The spheroids were harvested, fixed, and qualitatively evaluated by Safranin O/Fast Green (Waldeck, Münster, Germany) staining (Figure 2a) following established protocols [23].

For adipogenic differentiation, the cells underwent 14 days of differentiation in adipogenic differentiation medium (DMEM high glucose (Thermo Fisher Scientific), 10% fetal calf serum (FCS) (Thermo Fisher Scientific), 0.1 IE/mL insulin glargin (Sanofi-Aventis, Frankfurt am Main, Germany), 0.2 mM indomethacin, 0.5 mM isobutyl methylxanthine, 1 μM dexamethasone (all Sigma-Aldrich), penicillin/streptomycin 100 mg/L (Merck), and amphotericin B 2.5 mg/L (Merck). After 14 days, cells were stained by Oilred-O, and counterstained by haemalaun working solution (both Waldeck) to qualitatively assess adipogenic differentiation by detection of red-colored fatty vacuoles (Figure 2b) [23].

### 4.3. Osteogenic Differentiation

MSC were expanded to passage 2 prior to osteogenic differentiation. 35,000 cells each were transferred into one well of 24-well plates (Thermo Fisher Scientific) and differentiated using 500 µL osteogenic differentiation medium (ODM) (25 mM DMEM high-glucose with L-glutamate (Thermo Fisher Scientific), 10% FCS (Thermo Fisher Scientific), penicillin/streptomycin 100 mg/L (Merck), amphotericin B 2.5 mg/L (Merck), dexamethasone 0.1 µM (Sigma-Aldrich, Steinheim, Germany), ascorbic acid-2-phosphate 2.5 mg/L (Sigma-Aldrich), and beta glycerophosphate 10 mM (Merck) per well. The incubation was performed under standard cell-culture conditions in an incubator (HeraCell 240i Incubator, Thermo Fisher Scientific) at 37 °C, 21% O_2_, and 5% CO_2_.

IGF-1 (R&D Systems, Wiesbaden, Germany) and BMP-7 (Olympus Biotech Europe, Lyon, France) were added to the ODM in concentrations of 100 ng/mL, 200 ng/mL, 400 ng/mL, 800 ng/mL, 1600 ng/mL, and 6400 ng/mL. Concentrations were set according to concentrations used in literature previously, supplemented by very high concentrations to determine a possible ceiling effect [12,23]. The stimulated groups were compared to a control group where no differentiation factors were added to the ODM.

The cells were harvested after 7 and 14 days in culture. Osteogenic differentiation was quantified indirectly by Alizarin Red staining (Waldeck, Münster, Germany) (Figure 2c) and ALP activity measurement, as described below. All measurements were carried out in both biological and technical duplicates, meaning each combination of donor, time point, cell type, and concentration of differentiation factor was established in two separate wells (biological duplicates), and two samples from each well were measured (technical duplicates). The four resulting values were averaged to form one value that was then used for statistical analysis.

### 4.4. Quantification of Osteogenic Differentiation

Osteogenic differentiation was measured by quantification of extracellular calcium deposit by Alizarin Red staining, as well as by evaluation of ALP activity normalized to total cellular protein, as published previously [23]. The ALP activity correlates with the osteogenic differentiation of cells, as it is produced and released by osteoblasts and osteoblast-like-cells [23]. Osteoblasts deposit calcium within an organized extracellular surrounding: Alizarin Red staining is able to determine the amount of calcium incorporated in the extracellular matrix, being another maker for early osteogenic differentiation [23].

In short, the MSC were fixated in 70% ethanol overnight, followed by staining with Alizarin Red (Waldeck). The stained calcium deposit was then solved by addition of 10% hexadecylpyridinium chloride (Merck), causing a turnover to violet color during the process. Extinction at 570 nm was photometrically determined, directly correlating with the amount of dissolved calcium within the solution (Figure 2c).

For ALP activity measurement, MSC underwent lysis in 1% triton (Sigma-Aldrich, Steinheim, Germany) buffer. A solution of para-Nitrophenylphosphate (PNPP; Sigma-Aldrich, Steinheim, Germany) was then added to the lysate (final concentration of para-Nitrophenylphosphate in the incubation solution was 500 µg/mL). ALP converts para-Nitrophenylphosphate into para-Nitrophenol (PNP), causing a change of lysate color to yellow in correlation with ALP activity. After 90 min, photometric measurement was performed at 405/490 nm to quantify this change (and the corresponding amount of PNP now in the solution) and thus assess the underlying activity of ALP. For proper quantification, ALP activity was normalized to total cellular protein content, assessed by Micro BCA™ Protein Assay Reagent Kit (Thermo Fisher Scientific) according to the manufacturer’s instructions. The resulting unit of ALP activity is ng PNP/ng Protein/mL (of lysate)/min, measuring the amount of product (PNP, in ng) converted from substrate (PNPP) by ALP per minute in a standardized volume of cell lysate (in mL) that is normalized to the cellular protein content (in ng) in order to standardize cellular conditions and properties: More cells would exhibit more ALP activity compared to a smaller number of cells. Therefore, since the protein production of the cells correlates with the cell number and general metabolic activity, ALP-activity is normalized to total cellular protein.

### 4.5. Statistics

Statistical analysis was performed with SPSS Statistics 22 (IBM, Armonk, NY, USA) using the Mann-Whitney-*U* test for comparing stimulation with IGF-1 to stimulation with BMP-7. To compare the individual concentrations with the corresponding unstimulated control group, the Wilcoxon signed-rank test for paired samples was used, as the addition of a stimulation factor represented the intervention in this case. *p*-values of <0.05 were regarded as statistically significant for both tests. Graphics were made using GraphPad Prism 5 (Version 5.01; GraphPad Software, La Jolla, CA, USA).

## 5. Conclusions

The results obtained in this study indicate that the application of IGF-1 in concentrations of 400–800 ng/mL should be considered as a valuable option to improve bone formation and osteogenic differentiation of MSC. The subtype of MSC and the way of application must be considered when it comes to the use of growth factors. Optimal concentrations and continuous release of growth factors is necessary to enhance osteogenic differentiation of BMSC and RMSC. Further understanding of the cellular characteristics is mandatory to understand the exact interaction mechanisms of cells and differentiation factors. The combination of multiple aspects of bone defect treatment should be taken into consideration to develop new approaches in bone defect therapy, for example, by integration of differentiation factors into bone substitutes or other carriers. In this way, controlled and constant release kinetics with a defined concentration can potentially be achieved, making the use of differentiation factors more efficient. The results of this study reveal that IGF-1 is able to improve osteogenic differentiation in a very early stage, even in low concentrations, and therefore should be considered for further studies and applications.

## Figures and Tables

**Table 1:** Results of flow cytometry analysis.

MSC-Subtype	CD90	CD73	CD105	Negative Control
BMSC	95.23% (0.88%)	99.97% (0.01%)	99.88% (0.05%)	0.51% (0.12%)
RMSC	96.49% (0.93%)	99.99% (<0.01%)	99.92% (0.04%)	0.63% (0.11%)

The table shows the mean percentage of cells that displayed the surface characteristics (cluster of differentiation—CD) determined necessary for mesenchymal stem cells (MSC). Cells have to be >95% positive for CD90, CD73, and CD105 and <2% positive for CD14, CD20, CD34, and CD45 (summarized as “Negative Control”). The standard error of the mean is shown in parentheses behind each value. All measurements were carried out in both biological and technical duplicates.

## Abbreviations

ALP	Alkaline phosphatase
ATSC	Adipose tissue derived mesenchymal stem cells
BMP-7	Bone morphogenetic protein 7
BMSC	Mesenchymal stem cells harvested via iliac crest bone marrow aspiration
BSA	Bovine serum albumin
CD	Cluster of differentiation
DMEM	Dulbecco’s modified eagle medium
FCS	Fetal calf serum
IGF-1	Insulin-like growth factor 1
MSC	human mesenchymal stem cells
ODM	Osteogenic differentiation medium
PNP	Para-Nitrophenol
PNPP	Para-Nitrophenylphosphate
RMSC	Mesenchymal stem cells harvested via Reamer-Irrigator-Aspirator
RUNX2	Runt-related transcription factor 2
TGF-β1	Transforming growth factor β1
